# Impact of Different [Tc(N)PNP]-Scaffolds on the Biological Properties of the Small cRGDfK Peptide: Synthesis, In Vitro and In Vivo Evaluations

**DOI:** 10.3390/molecules27082548

**Published:** 2022-04-14

**Authors:** Nicola Salvarese, Debora Carpanese, Laura Meléndez-Alafort, Laura De Nardo, Andrea Calderan, Barbara Biondi, Paolo Ruzza, Antonio Rosato, Cristina Bolzati

**Affiliations:** 1 Institute of Condensed Matter Chemistry and Technologies for Energy ICMATE-CNR, Corso Stati Uniti, 4, 35127 Padova, Italy; nicola.salvarese@cnr.it; 2 Immunology and Molecular Oncology Unit, Veneto Institute of Oncology IOV-IRCCS, Via Gattamelata 64, 35138 Padova, Italy; debora.carpanese@iov.veneto.it (D.C.); laura.melendezalafort@iov.veneto.it (L.M.-A.); antonio.rosato@unipd.it (A.R.); 3 Department of Physics and Astronomy, University of Padova, Via Marzolo 8, 35131 Padova, Italy; laura.denardo@unipd.it; 4 Institute of Biomolecular Chemistry of CNR, Padova Unit, Via Marzolo 1, 35131 Padova, Italy; andrea.calderan@cnr.it (A.C.); barbara.biondi@cnr.it (B.B.); paolo.ruzza@cnr.it (P.R.); 5 Department of Surgery, Oncology and Gastroenterology, University of Padova, Via Gattamelata 64, 35138 Padova, Italy

**Keywords:** Tc-99m, α_v_β_3_ integrin, RGD peptides, imaging, targeting molecules

## Abstract

**Background**: The [^99m^Tc][Tc(N)(PNP)] system, where PNP is a bisphosphinoamine, is an interesting platform for the development of tumor ‘*receptor-specific*’ agents. Here, we compared the reactivity and impact of three [Tc(N)(PNP)] frameworks on the stability, receptor targeting properties, biodistribution, and metabolism of the corresponding [^99m^Tc][Tc(N)(PNP)]-tagged cRGDfK peptide to determine the best performing agent and to select the framework useful for the preparation of [^99m^Tc][Tc(N)(PNP)]-housing molecular targeting agents. **Methods:** cRGDfK pentapeptide was conjugated to Cys and labeled with each [Tc(N)(PNP)] framework. Radioconjugates were assessed for their lipophilicity, stability, in vitro and in vivo targeting properties, and performance. **Results:** All compounds were equally synthetically accessible and easy to purify (RCY ≥ 95%). The main influences of the synthon on the targeting peptide were observed in in vitro cell binding and in vivo. **Conclusions:** The variation in the substituents on the phosphorus atoms of the PNP enables a fine tuning of the biological features of the radioconjugates. *ws*[^99m^Tc][Tc(N)(PNP3OH)]– and [^99m^Tc][Tc(N)(PNP3)]– are better performing synthons in terms of labeling efficiency and in vivo performance than the [^99m^Tc][Tc(N)(PNP43)] framework and are therefore more suitable for further radiopharmaceutical purposes. Furthermore, the good labeling properties of the *ws*[^99m^Tc][Tc(N)(PNP3OH)]– framework can be exploited to extend this technology to the labeling of temperature-sensitive biomolecules suitable for SPECT imaging.

## 1. Introduction

In spite of the wide availability of PET radionuclides and the plentitude of new PET tracers that have sharply reduced the number of new SPECT agents introduced into clinical practice, [^99m^Tc]-technetium-tagging remains an attractive approach in the preparation of radiolabeled peptides for SPECT imaging [1,2]. Indeed, the basic research in ^99m^Tc-radiopharmaceuticals has been able to keep abreast with the trends in molecular imaging, particularly developing effective ^99m^Tc-analogues of ^68^Ga-radiopharmaceuticals.

Thanks to its ideal emission properties (E_γ_ = 142 keV; t_½_ = 6.02 h), easy availability at low cost, and rich and versatile coordination chemistry, ^99m^Tc has the potential for more widespread applications with respect to other less available and more expensive radionuclides. To further increase its convenience, ^99m^Tc-biotargeting agents could be prepared via commercially available lyophilized cold kits.

Over the past years, a number of different radiolabeled peptides used for SPECT and PET noninvasive preclinical/clinical imaging studies have been reported [3,4,5,6,7]. For all these compounds, the radionuclide/radiometal and the chelating system are found to influence their distribution profile as well as the image quality [8].

Among the different approaches used for the preparation of Tc-tagged tumor target-specific radiopharmaceuticals, over the past decades, we have described the synthesis and biological evaluation of a series of discrete asymmetrical [^99m^Tc][Tc(N)(PNP)]-housed nitride compounds, where PNP is a bulky bisphosphinoamine, as an interesting paradigm of synthesis for the design and development of metal-essential and tumor ‘*receptor-specific*’ agents [9,10,11]. The chemical and reactivity features of the system along with the strengths and weaknesses of the usage of this technology have been recently reviewed [12]. The peculiarity of this technology is the high chemical flexibility, which permits the fine-tuning of their physical and chemical properties (lipophilicity, shape, size, and charge) through the independent variation in the PNP substituents (both at P and N atoms) and/or of the co-ligand choosing between sulphur-contained bi-anionic or monoanionic chelators ([S^S]^2−^, [O^S]^2−^, [H_2_N^S]^−^). Therefore, chelation systems can be rigorously selected to obtain the right balance between stability, charge, and lipophilicity of the complexes, thus improving their pharmacokinetics and making possible the modulation of their biological properties; an example of this is given by the studies carried out in the development of a series of myocardial perfusion imaging agents [13,14,15].

In addition, the chemical and reactivity properties of the activated [Tc(N)Cl_2_(PNP)] intermediate complex were strongly influenced by the chemical nature and by the N-nucleophilicity of the bisphosphinoamine [16,17,18]. In particular, the insertion of low encumbering and hydrophilic substituents (e.g., -CH_2_OH) on the P atoms of PNP has a marked impact on its reactivity properties, improving the rate of formation of both the activated intermediate and the final heteroleptic complexes. The quantitative formation of the [^99m^Tc][Tc(N)(PNP)]-tagged molecules is reached after 30 min incubation at room temperature by using a co-ligand amount in the range 10^−5^–10^−7^ M, in physiological solution, equally applying a two-step or one-step route [19], paving the way for the [^99m^Tc][Tc(N)(PNP)]-labeling of temperature-sensitive active biomolecules.

By considering tumor target-specific agents, it is well known that their pharmacokinetics and target accumulation are due to ‘specific’ and ‘non-specific’ interactions, which most likely rely on the whole physico-chemical properties of the radiolabeled molecule (i.e., molecular weight, overall charge, lipophilicity). Such features are in general modulated by varying the nature of the chelator as well as the radiometal, each of which may have an impact on the net charge of the conjugate-complex, by adding pharmacokinetic modulators (PKM; e.g., PEG, carbohydrate, etc.) and by modifying the peptide chain.

As outlined above, exploiting the [^99m^Tc][Tc(N)(PNP)] technology, both molecular weight and lipophilicity of the radiolabeled peptides can be easily modified by varying the [^99m^Tc][Tc(N)(PNP)] scaffold, switching the substituents on the phosphorus from bulky aryl (C_6_H_5_-) or alkoxy-alkyl (-CH_2_CH_2_CH_2_OCH_2_CH_3_; -CH_2_CH_2_CH_2_OCH_3_) to tiny and more hydrophilic groups (i.e., -CH_3_, -CH_2_OH). For such compounds, in principle, the reduction in the scaffold size and lipophilicity may positively affect the biological properties of the targeting vector, increasing the specificity against the receptor site and its pharmacokinetics. On the other hand, it might also affect the overall chemical and kinetic stability of the final heteroleptic complex [19].

With the aim of determining the best performing framework useful for the preparation of molecular targeting agents, this study was addressed to evaluate the impact of different [^99m^Tc][Tc(N)(PNP)] moieties on the synthesis and biological fate of a small peptide. The cyclo(Arg-Gly-Asp-_D_Phe-Lys) pentapeptide (cRGDfK) was selected as a molecular vector [20,21,22]. Due to its small size, the influence of the radioactive chelate, depending on the nature and molecular size of the building block, will be evident [23,24]. cRGDfK is known to target α_v_β_3_ and α_v_β_5_ integrins, which are involved in pathophysiological processes such as tumor metastasis, neoangiogenesis, osteoporosis, restenosis, and inflammation [20,21,22,25,26,27], and it can be easily conjugated to the ε-amino group of the Lys residue with chelating moieties, directly or via an appropriate PKM [28]. RGD-based agents have already been investigated with a large variety in the RGD-containing unit [29], PKM item, and labeling system, including nuclides [30,31,32,33,34]. In general, the corresponding radiolabeled compound disclosed a high in vivo stability and high-binding affinity to integrins overexpressed on different tumors.

**Figure 1:**
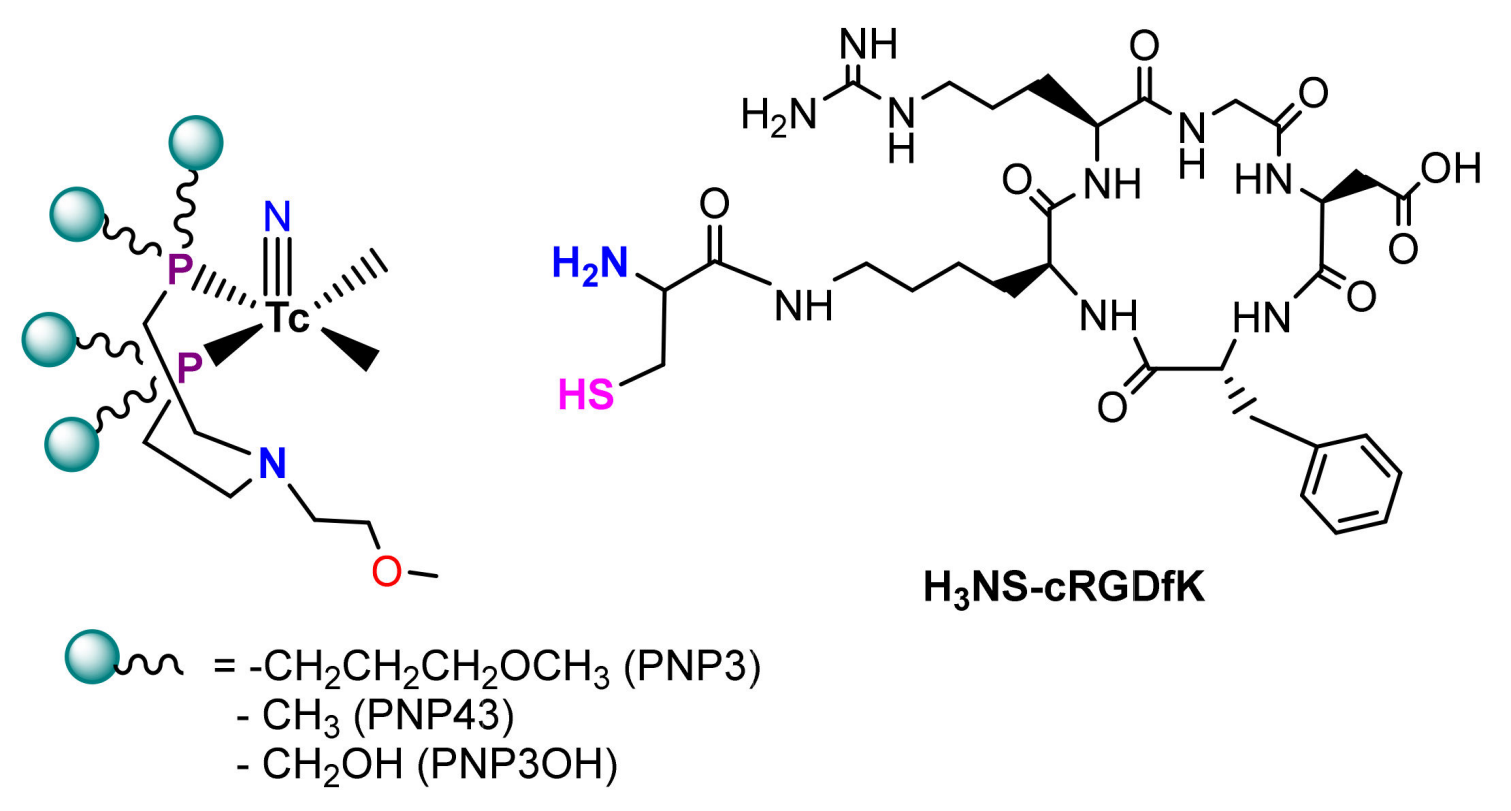
Left side: schematic drawing of the [^99m^Tc][Tc(N)(PNP)] synthons: PNP act as bidentate ligands, whereas the remaining two coordinative positions around the [Tc≡N]^2+^ core are free for the coordination with a cysteine conjugated biomolecule. Right side: the cysteine-conjugated cRGDfK pentapeptide used in our experiments.

Herein, we report the synthesis and biological evaluation of three different [^99m^Tc][Tc(N)(PNP)]-tagged cRGDfK, where PNP was alternatively: (1) the widely used *N*,*N*-bis[(dimethoxypropylphosphino)ethyl]methoxyethylamine, PNP3, selected for its bulky properties (for **^99m^Tc1**); (2) the lower encumbered *N*,*N*-[bis(dimethylphosphino)ethyl]methoxyethylamine, PNP43, selected to minimize any possible interaction between the building block and the bioactive molecule (for **^99m^Tc2**); (3) the water-soluble *N*,*N*-bis[(di-hydroxymethylphosphinoethyl)]methoxyethylamine, PNP3OH, selected to reduce the lipophilic properties of the building block (for **^99m^Tc3**). Notably, PNP43 has been lately applied to the labeling of the selective α_v_β3 antagonist RGDechi-peptide. Although the tracer displayed good pharmacokinetics and high αvβ_3_ selectivity and specificity, it suffers from a low absolute tumor uptake, thus needing an improvement [11]. Additionally, PNP3OH was recently investigated for the possibility of labeling temperature-sensitive molecules [19]. The chemical structure of [^99m^Tc][Tc(N)(PNP)] scaffolds and of H_3_NS-cRGDfK used in this investigation are sketched in Figure 1.

The peptide was modified at the ε-amino group of the Lys residue anchoring site by direct conjugation of the carboxyl group of cysteine (H_3_NS-cRGDfK) as the chelating moiety, thus leaving the [NH_2_,S^−^] pair of donor atoms free for coordination to yield the corresponding monocationic [^99m^Tc][Tc(N)(H_2_NS-cRGDfK)(PNPn)]^+^ species.

The radiocompounds were characterized by means of HPLC chromatography, determination of partition coefficients (Log P), and assessment of their inertness under conditions relevant to in vivo application.

Pharmacological evaluations of [^99m^Tc][Tc(N)(H_2_NS-cRGDfK)(PNPn)]^+^ complexes were assessed through competitive cell binding and internalization experiments performed on α_v_β_3_ (M21) and α_v_β_5_ (MCF7)-positive and integrin-negative (M21L) cell lines, as well through biodistribution studies. Pharmacokinetics of the conjugated complexes were evaluated in healthy female Sprague Dawley rats, analyzing the data by CoKiMo software [35]. Athymic nude mice bearing α_v_β_3_-positive and α_v_β_3_-negative melanoma tumors were used as the in vivo model for tumor distribution and metabolism studies. Data were validated by using the corresponding [^99m^Tc]Tc-HYNIC-cRGDfK as a control.

## 2. Results

### 2.1. Peptide Synthesis

The N-functionalized cysteine–RGD peptide was prepared using manual solid-phase peptide synthesis starting from Fmoc-L-Asp(Wang-Resin)-OAll. Peptide was assembled using the Fmoc/TBTU chemistry in 0.06 mmolar scale. TBTU/HOBt activation employed a three-fold molar excess (0.24 mmolar) of Fmoc amino acids in DMF solution for each coupling cycle. After assembly of the Fmoc-D-Phe-Lys(ivDde)-Arg(Pbf)-Gly-Asp(Wang-Resin)-OAll pentapeptide, the C-terminal -OAll protecting group was removed according to the procedure described by Grieco et al. [36] using Pd(PPh_3_)_4_/PhSiH_3_ in DCM as reagent. Successively, the N-terminal Fmoc-protecting group was removed by standard protocol, and the peptide was cyclized on-resin using TBTU/HOBt as coupling reagent. The head-to-tail cyclization on solid phase is advantageous rather than in solution. A pseudo-dilution phenomenon is achieved by having the linear peptide attached to a polymeric support, and intramolecular cyclization is favored over intermolecular because the solid support provides a large distance between the chains [37] and references therein. Furthermore, cyclization can be driven to completion using an excess of reagents that can be easily removed by filtration and washing. Finally, the ivDde-protecting group was removed from Lys side chain by treatment with a diluted solution of hydrazine (2%) in DMF to allow the coupling to the carboxyl group of a cysteine residue, thus leaving the [NH_2_,S^−^] pair of donor atoms available for coordination to the [^99m^Tc(N)(PNPn)]-building block. After RP-HPLC purification, the peptide was obtained in good yield with a purity of 95%. The identity was confirmed by electrospray ionization mass spectrometry (see Figure S1 in Supplementary Materials).

### 2.2. Radiosyntheses

**Chart 1:**
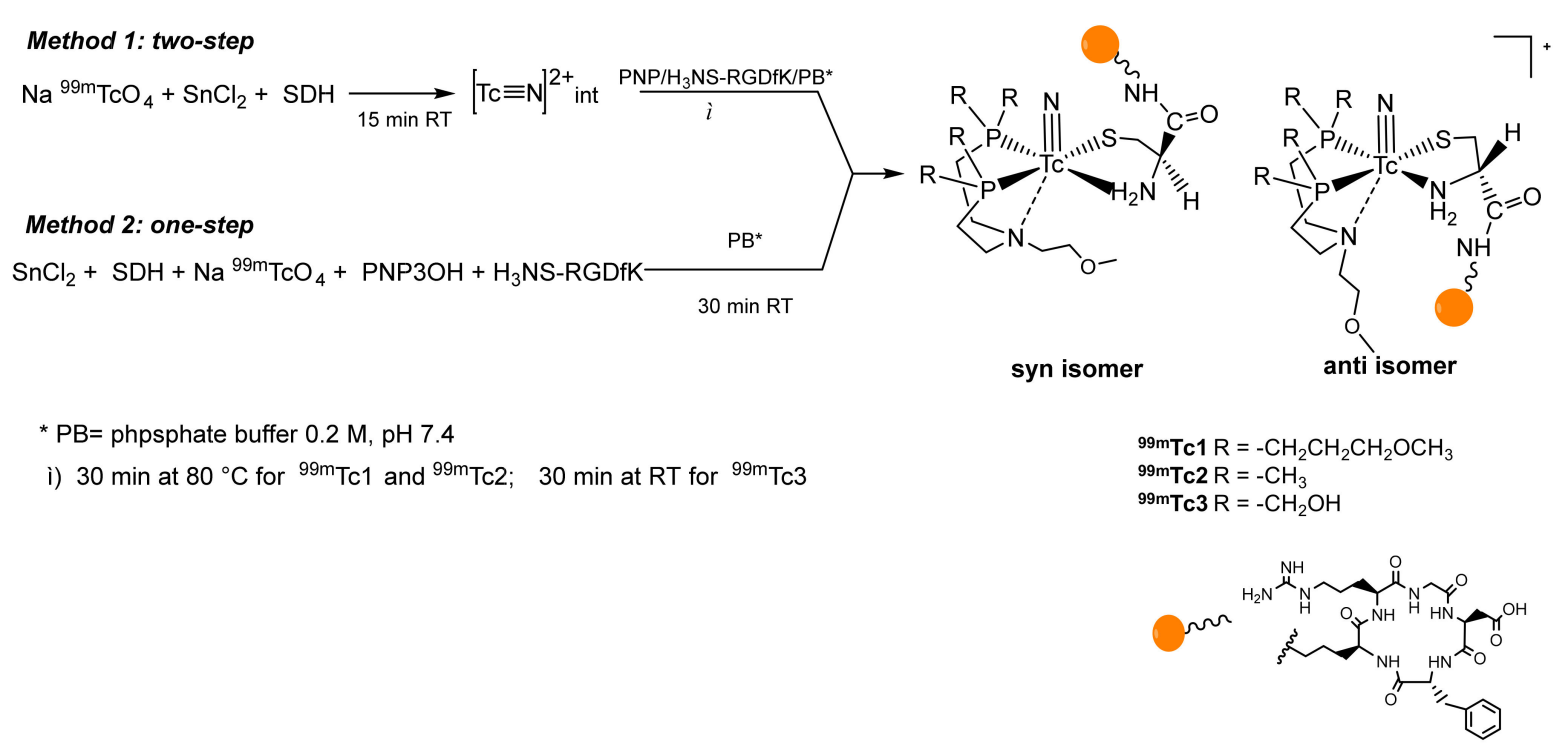
Pathways of synthesis of [^99m^Tc(N)(H_2_NS-cRGDfK)(PNP)]^+^ compounds along with a drawing of their *syn* and *anti*-isomers.

Monocationic [^99m^Tc][Tc(N)(H_2_NS-cRGDfK)(PNPn)]^+^ (**^99m^Tc1–3**) were efficiently prepared at neutral pH, starting from [^99m^Tc]NaTcO_4_ according to the well-established two-step or one-step pathway of synthesis (Chart 1). In all cases, the reaction progress was followed by chromatographic techniques.

For **^99m^Tc1** and **^99m^Tc2**, the reactions were ended after 30 min of heating at 80 °C, while for **^99m^Tc3**, radiosynthesis was completed after 30 min of incubation at room temperature. Neither intermediate species (assigned as [^99m^Tc][Tc(N)]_int_ or [^99m^Tc][Tc(N)(X_2_)(PNP)], X = -Cl, -OH, H_2_O) nor radiolabeled byproducts were detected in the reaction mixture, invariably of the PNP type used.

The RCY of **^99m^Tc1–3**, along with their chromatographic properties are reported in Table 1.

Chromatograms of the ^99m^Tc(N) complexes showed the characteristic two peaks of different intensity, which are the *syn* and *anti*-isomers of [^99m^Tc(N)(H_2_NS-cRGDfK)(PNP)]^+^ compounds resulting from the alignment of the substituent anchored to the carboxyl group of the cysteine chelator with respect to the Tc≡N multiple bond. Studies on various [^99m^Tc][Tc(N)(PNP)]-tagged NH_2_-terminal cysteine-derivate peptides showed a close relationship between the two isomeric forms, for which a reversible and temperature-dependent isomeric conversion was proved, reaching the a/b equilibrium of 70:30 [10]. In addition, receptor affinity studies, carried out with the avidin/biotin system as a model, disclosed that when a five-term chain is placed between the Cys-chelator and the bioactive effector, the two isomers keep almost the same target affinity [38]. Hence, the mixture of *a* and *b* forms as attained from the standard preparation was used upon the solid-phase extraction and purification for distribution coefficient (Log P) determination, and for in vitro and animal studies.

*N*,*N*-bis(di-hydroxymethylenphosphinoethyl)methoxyethylamine (PNP3OH) was obtained as phosphonium salt PNP3OH•HCl, which was rapidly and quantitatively converted into PNP3OH free form under basic or neutral pH after the addition of carbonate buffer 0.5 M pH 9 or phosphate buffer (PB) 0.2 M pH 7.4, respectively [19]. To assess the possibility of using the highly stable PNP3OH•HCl in radiosyntheses for the in situ generation of PNP3OH, a series of pH-dependent preparations was set up.

**Figure 2:**
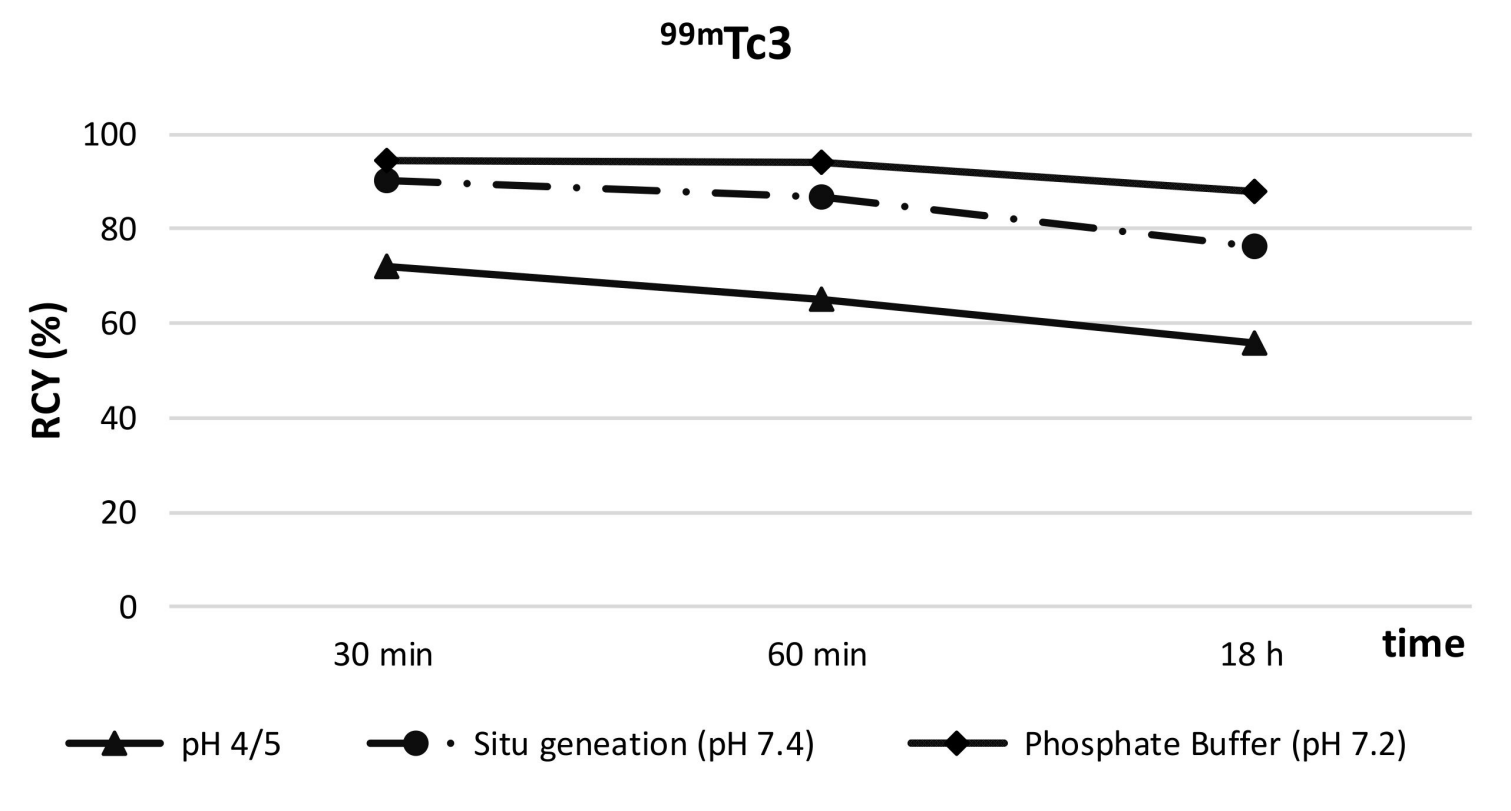
Over time variation in RCY(%) of ^99m^Tc3 as function of the pH.

Figure 2 shows the variation in % RCY of **^99m^Tc3** over time (30 min vs. 18 h) under the different conditions. PNP3OH•HCl was employed at autogenous pH (4/5) or converted in situ into the corresponding free PNP3OH by the addition of PB 0.2 M pH 7.4. Alternatively, free PNP3OH was directly used. This latter was obtained by dissolving PNP3OH•HCl in PB before the use. At 30 min, the highest RCYs were obtained with in situ generation or using the PNP3OH buffered solutions. The complexes were stable within 18 h in their reaction solution at pH 7.4.

**Figure 3:**
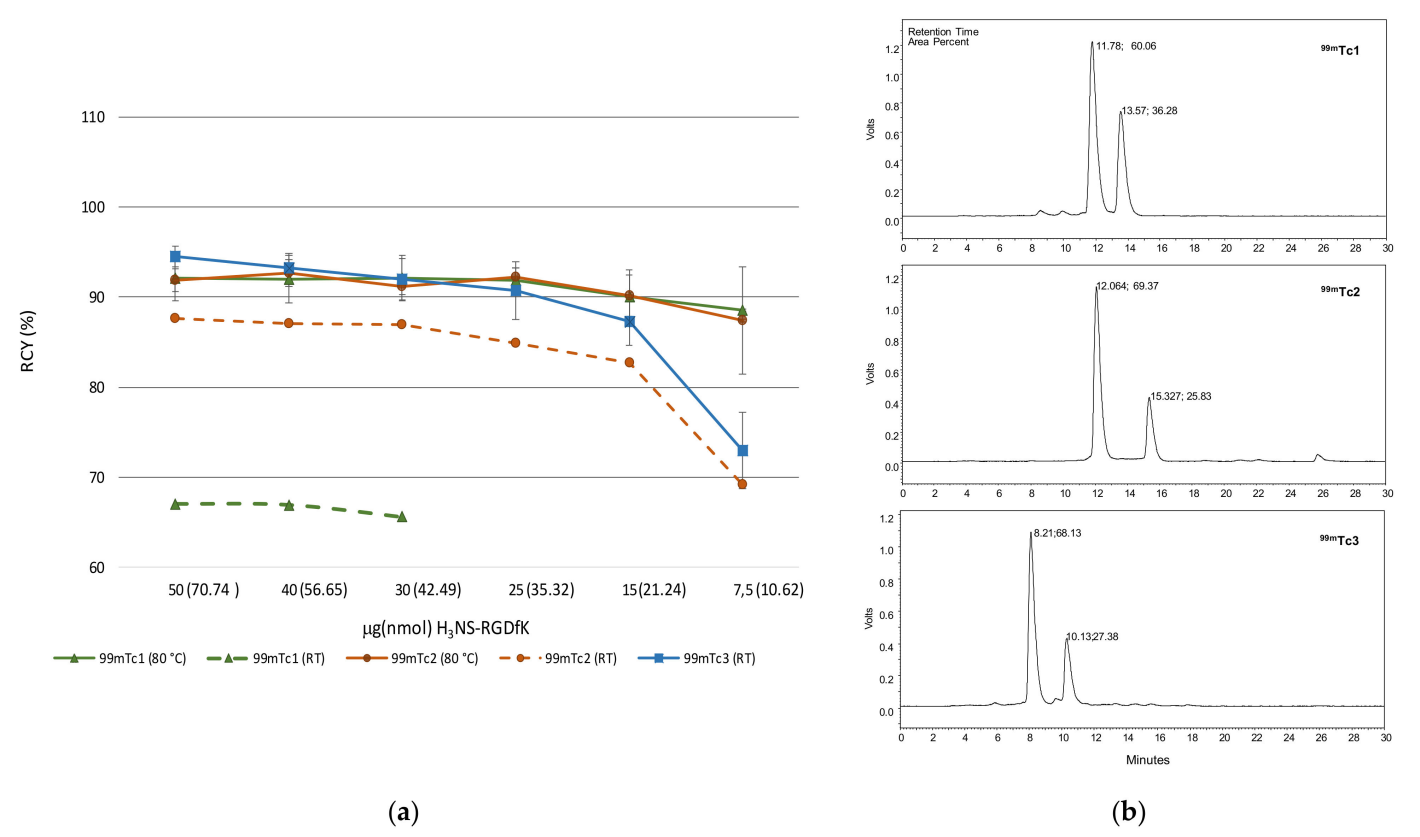
(**a**) Labeling efficiency; (**b**) chromatograms of the purified **^99m^Tc1–3** collected according to method 2 described in Table 1.

The radiolabeling efficiency of the H_3_NS-cRGDfK peptide was determined for each compound, adopting the following standardized labeling conditions: PNP amount, 9.94 × 10^−4^ mmol; final volume, 1.5 mL; incubation time, 30 min. The peptide amount was progressively decreased in the range: 0.50 mg (70.17 nmol)–0.075 mg (10.62 nmol). The RCY was determined by HPLC. Figure 3 shows the dependence of the complex RCY on the H_3_NS-cRGDfK amount along with the chromatograms of **^99m^Tc1–3**.

For **^99m^Tc1** and **^99m^Tc2**, the high RCY was achieved at 80 °C, with an amount of H_3_NS-cRGDfK in the range of 15–25 µg (1.41 × 10^−5^–2.3 × 10^−5^ mol/L). Lower temperature (RT) significantly reduced the RCY of **^99m^Tc1**, whereas the yield of **^99m^Tc2** still remained acceptable. The high RCY of **^99m^Tc3** was achieved at room temperature, using from 20 to 30 µg of H_3_NS-cRGDfK (3.35 × 10^−5^–7.07 × 10^−5^ mol/L), according to the previously reported data [19]. Using these amounts, at the end of radiosynthesis carried out with 2.22 GBq of starting activity, the effective specific activities of the complexes were estimated to be 94.04–56.42 for **^99m^Tc1** and **^99m^Tc2** and 72.10–49.11 for **^99m^Tc3**.

**^99m^Tc1–3** were solid phase extracted in order to maximize their effective specific activity and to eliminate the bis-phosphinoamine and the excess of unlabeled peptide, which could compete with the radiotracer for the target expressed in both tumor and healthy tissues. After the procedure, the purity of the radiopeptides, determined by HPLC chromatography (UV, radio), was more than 95% (Figure 3b). No evidence of both H_3_NS-cRGDfK and PNP ligands was observed in UV-HPLC chromatograms of the collected fraction.

To explore the impact of the PNP ligand on the lipophilicity of the complexes, Log Ps in a mixture of n-octanol and PBS were determined. Data, expressed as (activity concentration in n-octanol)/(activity concentration in water), are reported in Table 1. The lipophilicity decreased in the order **^99m^Tc1** > **^99m^Tc2** > **^99m^Tc3.** The lipophilic character of each compound correlated well with the corresponding HPLC retention time and protein binding behavior.

The inertness of the radioconjugates under conditions relevant for in vivo applications were assessed by evaluating the stability of **^99m^Tc1–3** in solution and after incubation (37 °C) with transchelating agents (glutathione and L-cysteine, 10–1 mM, pH 7.4).

The complexes were stable in the reaction solution for >12 h post-labeling and for 24 h after purification and dilution in injectable solutions. Stability experiments against small molecule challengers of the radiometal coordination sphere showed that **^99m^Tc1–3** were adequately stable. In particular, **^99m^Tc1** and **^99m^Tc2** were found to be unaffected (%RCP > 95%) by the large excess of glutathione and L-cysteine through 24 h. **^99m^Tc3** showed minimal RCP variation in the challenge reaction with GSH (% RCP = 73.33 ± 2.8 at 24 h), while a reduction in %RCP was observed after 24 h incubation with free L-cysteine 10 mM (% RCP = 27.31 ± 3.6). A significant lowering of complex transchelation rate was observed when challenge studies were carried out with Cys 1 mM.

### 2.3. Cell Studies

Cell uptake and internalization studies were performed on receptor-positive human melanoma M21 (expressing high levels of α_v_β_3_ integrin and moderate level of integrin α_v_β_5_), human breast cancer MCF7 (exclusively expressing α_v_β_5_), and receptor-negative human melanoma M21L cell lines [39]. The expression of α_v_β_3_ and α_v_β_5_ by cells was already evaluated by flow cytometry (Table 2) [11]. The stability of each radiolabeled peptide in the culture media before and after cell exposure was assessed. The compounds were stable and, importantly, no variation on the a-to-b isomeric ratio was detected after cell contact, suggesting that the two isomeric forms were equally recognized by the molecular target. **^99m^Tc**-HYNIC-cRGDfK peptide was used as the reference compound [23,40].

**Figure 4:**
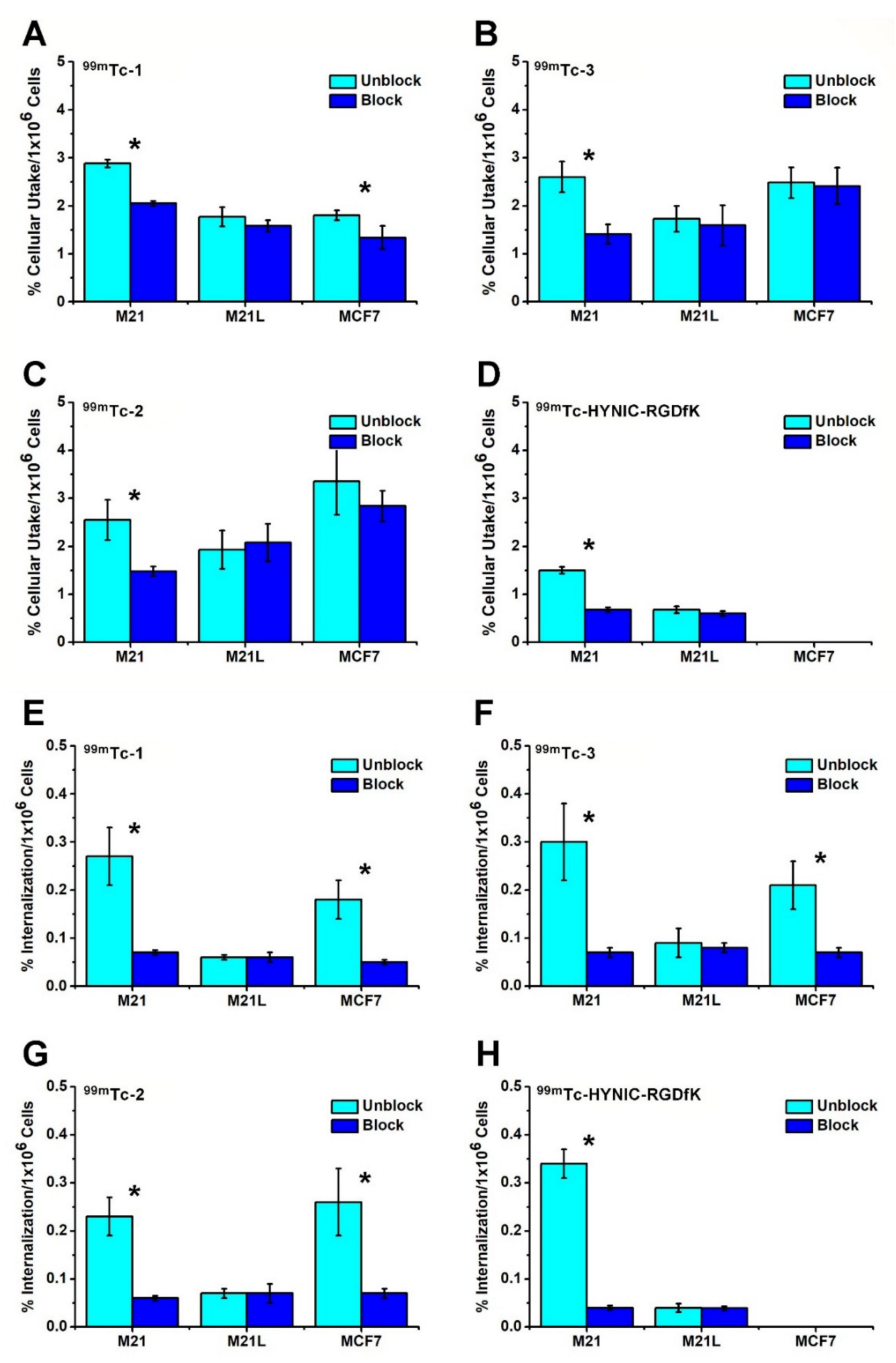
In vitro ^99m^Tc complexes cellular uptake (**A**–**D**) and internalization (**E**–**H**) in integrin-positive and integrin-negative tumor cell lines. * Statistically significant difference between block and unblock cells (*p* value ≤ 0.05).

Comparative cell uptake and internalization data are reported in Figure 4. The accumulation amounts of the complexes in receptor-positive M21 (α_v_β_3_) and MCF7 (α_v_β_5_) cells were compared with the unspecific uptake in M21 and MCF7 blocked with an excess of unlabeled cRGDfK pentapeptide, as well as the accumulation in receptor negative M21L and in M21L blocked cells. As regards human melanoma cell lines, **^99m^Tc1–3** showed a specific uptake (sum of surface bound and internalized radioactivity) in α_v_β_3_-positive cells, with values of 2.88 ± 0.09 for **^99m^Tc1**, 2.57 ± 0.42 for **^99m^Tc**2, and 2.60 ± 0.36 for **^99m^Tc**3. The uptake values were significantly lower when α_v_β_3_ receptors were blocked by an excess of cold cRGDfK (2.15 ± 0.17 for **^99m^Tc1**, 1.48 ± 0.12 for **^99m^Tc**2, and 1.41 ± 0.25 for **^99m^Tc**3) or in the negative M21L cell line (1.77 ± 0.20 for **^99m^Tc1**, 1.65 ± 0.40 for **^99m^Tc**2, and 1.73 ± 0.27 for**^99m^Tc**3), indicating that the uptake was receptor-mediated. The amounts of cell accumulation in M21 (unblocked and blocked) and in M21L of **^99m^Tc**-HYNIC-cRGDfK were 1.50 ± 0.08, 0.85 ± 0.08, and 1.30 ± 0.15, respectively—significantly lower than those of **^99m^Tc1–3**

The ratios between specific and non-specific uptake for **^99m^Tc1–3** ranged from 1.7 to 1.2, but when only the internalized radioactivity was considered, the specific/nonspecific ratios increased to 4.5 for **^99m^Tc1**, 4.29 for **^99m^Tc3**, and 3.28 for **^99m^Tc2**. For **^99m^Tc**-HYNIC-cRGDfK, the ratios were 1.15 and 6.6, respectively.

In MCF7 expressing α_v_β_5_ receptors, a cellular accumulation (sum surface bound and internalized radioactivity) ranging from 1.5 to 3 was detected. In this case, blocking studies revealed, with respect to M21 cells, only a minimal variation in the percentage of cellular accumulation of the compounds, indicating a high nonspecific binding to the cell membrane_._ Nevertheless, the internalization of all radioconjugates was strongly inhibited, with a reduction of 73% of the internalized activity, just further proving that internalization is a receptor-mediated process and that the compounds are recognized also by integrin α_v_β_5_.

### 2.4. Metabolism and In Vivo Studies

#### 2.4.1. In Vitro Metabolism

The in vivo distribution properties of a radiolabeled small peptide are determined by the size and type of the targeting molecule, the metal chelate (radiometal and chelating system), the overall molecular charge, lipophilicity, and protein-binding affinity, as well as by its resistance to the proteolytic degradation process.

Thus, to have some predictive indications of the metabolic outcome of our compounds, the binding to serum proteins and the enzymatic biotransformation of **^99m^Tc1–3** in blood and tissue homogenates were assessed in vitro by incubating the purified compounds at 37 °C in human and mouse sera as well as in murine liver and kidney homogenates. Complex extraction efficiency from the homogenate tissues was found to range from 80% to 90%.

When incubated with sera (37 °C, 18 h), the radioactivity bound to serum proteins was low (10–15%), irrespective of the hindrance exerted by the [^99m^Tc][Tc(N)(PNP)] moiety and of the hydrophilic character of the molecules. In all cases, after 4 h, the non-protein-bound radioactivity was identified as intact radiolabeled peptide (RCP > 90%); only a minor decomposition (<15%) caused by endogenous peptidases was found only after 18 h.

Moreover, RP-HPLC profiles collected over a period of 4 h after exposure in liver and kidney homogenates showed a high in vitro stability with minimal decomposition at longer incubation time.

**Figure 5:**
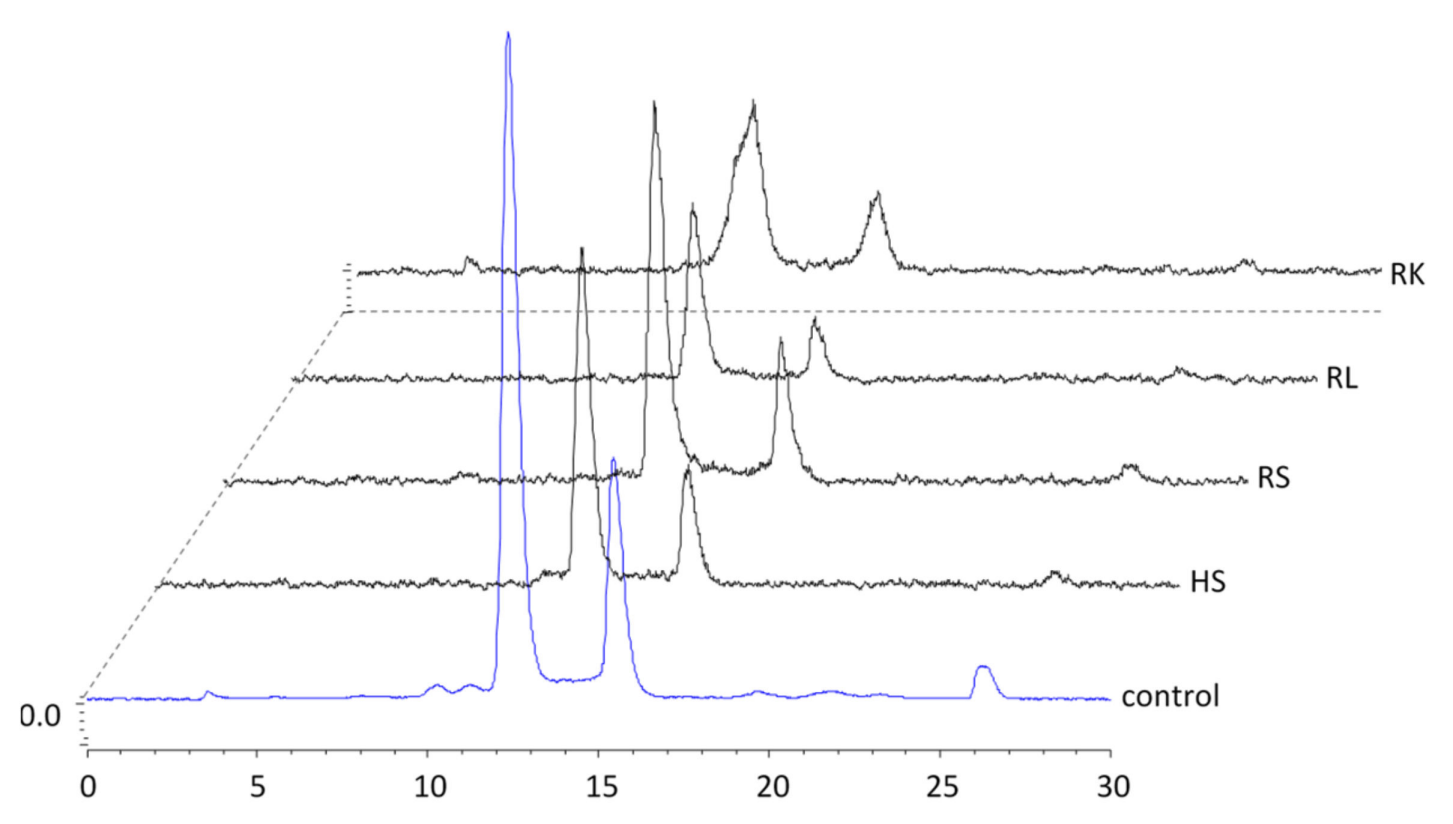
RP-HPLC of **^99m^Tc2** after incubation at 37 °C for 4 h in: (HS) human serum; (RS) rat serum; (RL) rat liver homogenate; (RK) rat kidney homogenate. The activity peaks of all samples were coincident with the control peaks, and no variations in the a-to-b isomeric ratio were observed, indicating that there is not a preference from peptidase towards one of the two isomers.

As an illustrative example, Figure 5 displays the RP-HPLC of **^99m^Tc2** post-incubation in human serum, rat serum, rat kidney, and liver homogenates. Stabilities of **^99m^Tc1, 3** were also previously reported and discussed [19].

#### 2.4.2. In Vivo Studies

*Pharmacokinetics and metabolism*. Table 3 shows the ex vivo biodistribution data obtained at four different time points after the administration of **^99m^Tc1–3** on healthy female Sprague Dawley rats, normalized as the percentage of injected activity per gram (%IA/g).

In general, all complexes disclosed a fast distribution to organs and metabolic sites, with a major uptake in the kidney, followed by the liver and intestine. This indicates that **^99m^Tc1–3** were excreted mainly by the renal pathway, although a small percentage of them was also eliminated through the liver and intestinal tract. In addition, a minimal and constant uptake in the stomach was detected for all complexes, proving the absence of ^99m^TcO_4_^−^ produced by the dissociation of the metal from the ^99m^Tc-conjugates and subsequent oxidation.

The activities in the other organs were lower than 1% IA/g after 0.5 h post injection for all compounds.

**Figure 6:**
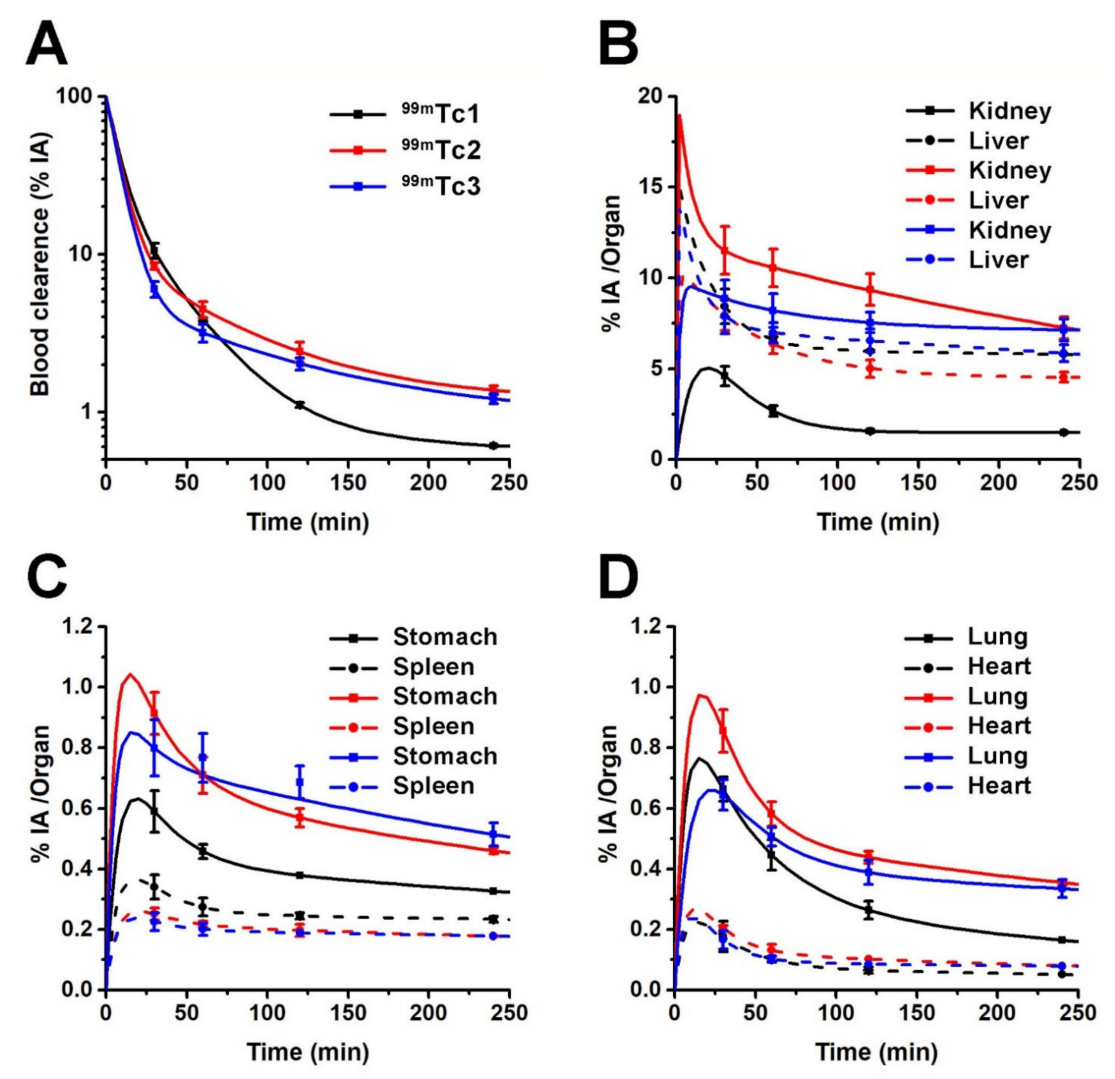
Time–activity curves in the main source organs: squares and circles show experimental data obtained from biodistribution studies after injection of 0.37 MBq of: **^99m^Tc1** (black), **^99m^Tc2** (red), and **^99m^Tc3** (blue), respectively. The four individual graphs represent: (**A**) blood, (**B**) kidney and liver, (**C**) stomach and spleen, and (**D**) lung and heart. The lines depict the calculated time–activity curves.

Figure 6 shows the time–activity curves of the main organs, obtained from the biodistribution data expressed as %IA/organ using CoKiMo software [35]. Results evidenced that the ^99m^Tc complexes presented a fast blood clearance, suggesting that the [Tc(N)(PNP)] scaffolds exert a modest impact on the protein-binding capability, and that translations of the radiometal to blood proteins did not occur in accordance with in vitro data (panel A). Organs such as stomach, spleen, lung, and heart displayed a fast washout of ^99m^Tc conjugates (panels C and D); in contrast, kidney and liver showed the slowest clearance (panel B). Among the complexes studied, **^99m^Tc1** outperformed the faster blood clearance and, consequently, the lower concentration in the main organs compared with **^99m^Tc2** and **^99m^Tc3**.

Table 4 shows the mean resident time (MRT) calculated with COKIMO software, by integration of the organ activity curves extrapolated until 30 h and corrected for physical decay using as input the mean of %IA/g obtained values for each time point. As expected, MRT values of liver and kidneys were much higher than the dose of stomach, spleen, lung, and heart.

The lower resident time in kidney of ^99m^Tc1 compared with ^99m^Tc2,3 complexes is due to the lower kidney uptake and faster clearance, as Figure 6B shows. This biological behavior might be explained by the different physical-chemical properties of the complex.

**Figure 7:**
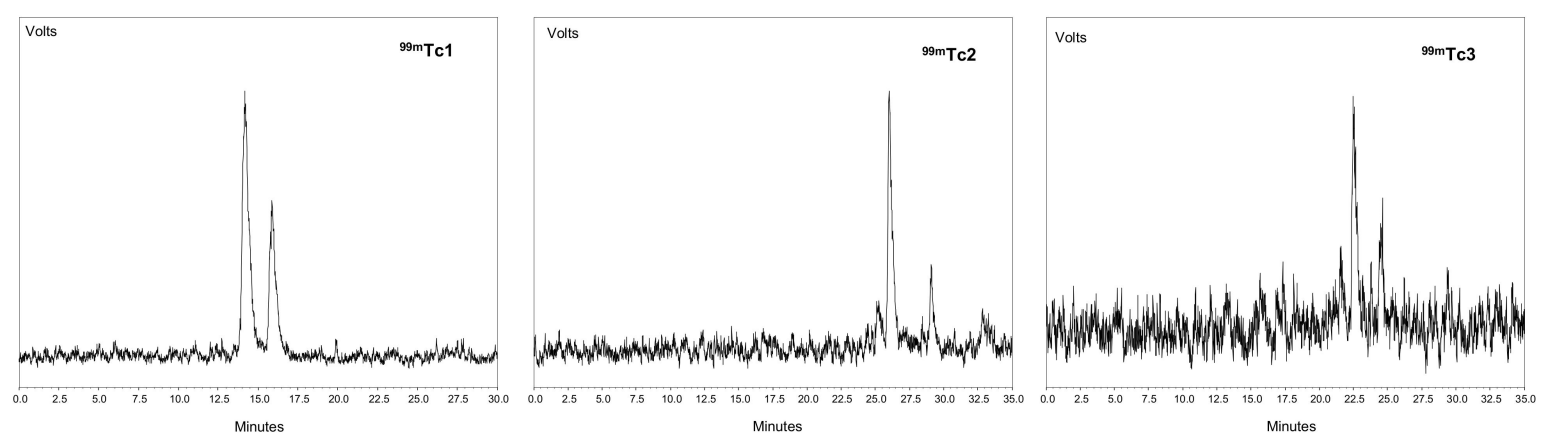
Chromatograms of the urine activity collected at 4h p.i. of **^99m^Tc1**, **^99m^Tc2**, **^99m^Tc3** collected by using the analysis conditions described in method 1 for **^99m^Tc1** and method 3 for **^99m^Tc2,3**.

As mentioned before, **^99m^Tc1–3** are mainly excreted from the renal system; therefore, samples of urine collected from rats at 2 and 4 h p.i. were analyzed by HPLC to study their in vivo stability (Figure 7). The complexes remained intact over 4 h p.i., underlining their high stability in vivo.

**In vivo tumor uptake**. The in vivo integrin targeting properties of the [^99m^TcN(PNP)]-labeled peptide were evaluated in M21 (integrin-positive) and M21L (integrin-negative) melanoma-bearing mice by comparing the percentage of tumor uptake of the three compounds in tumors and the respective target-to-non-target ratios. The organ uptake expressed as %IA/g and the tumor-to-background ratios at 2 h p.i. are summarized in Table 5. Biodistribution data outlined some differences in organ uptake compared with those attained in rats at the same injection time because the rate of metabolism is different in each animal species. However, in principle, a comparable biological behavior was found; the radioconjugates presented a low circulation/background activity as a result of a fast blood clearance and showed the highest concentration in the kidney, followed by liver and intestine. In all cases, the activity in M21 xenografts was significantly higher than that calculated for M21L control tumors, suggesting that the tracer accumulates more preferentially in receptor-positive tumor masses. **^99m^Tc3** exhibited the highest tumor uptake: 3.9 ± 0.7, followed by **^99m^Tc1** (3.2 ± 0.6) and **^99m^Tc2** (1.1 ± 0.2). Consequently, **^99m^Tc3** outperformed the best tumor-to-organ and tumor-to-background ratios (see Table 5), with a calculated M21/M21L ratio of 4.17, indicating that this compound is the best performing in vivo for receptor-positive tumor accumulation. In addition, favorable target-to-background ratios were also reached (e.g., M21/blood = 146 M21/muscle 21.9 at 120 p.i.). The high renal uptake of radiotracers is partially due to their rate of excretion as well as to a specific uptake by integrins expression in the kidneys; for **^99m^Tc3,** the M21-to-kidney ratio was 1.37.

Results were compared with those reported in the literature for ^99m^Tc-HYNIC-RGD on the same animal model and collected at 1 and 4 h p.i. [23,40]. Although the experimental end point of [^99m^TcN(PNP)]-labeled peptide was fixed at 2 h p.i., the %IA/g values achieved, in general, well matched those of ^99m^Tc-HYNIC-RGD collected at 4 h p.i. **^99m^Tc3** seems to be the best performing radiopeptide of the series, followed by **^99m^Tc1** and **^99m^Tc2**. Actually, it displays a higher tumor uptake, better distribution profile, and superior target-to-non-target ratio at earlier injection time when compared with the ^99m^Tc-HYNIC-RGD used as reference compound.

## 3. Discussion

In the search of effective methods for preparing metal-based target-specific radiopharmaceuticals, our group has been focusing on the development of [M(N)(PNP)]-tagged mixed-ligand complexes. Radioconjugates containing the [M(N)(PNP)] framework attached to biomolecules including serotonin and benzodiazepine receptor ligands, fatty acids, and peptides have been investigated [41,42,43,44,45]. These studies were mainly based on the use of aryl or bulky alkoxy alkyl PNP type ligands responsible for a primarily lipophilic nature of the moiety. This is an inherent characteristic since the metal is well shielded by the arrangement and by the electronic properties of the PNP ligand [12,16,17].

Exploiting the high chemical flexibility of this system, in this investigation, we compared the reactivity and the impact of the physical-chemical properties of three [Tc(N)(PNP)] frameworks on the stability, α_v_β_3_ and α_v_β_5_ targeting properties, biodistribution, and metabolism of the corresponding [^99m^Tc][Tc(N)(PNP)]-tagged cRGDfK peptides to determine the best performing framework useful for the further preparation of molecular targeting agents.

Complexes including both less-encumbered and hydrophilic PNP ligands were thermodynamically accessible in the sense that they formed in good yield, could be characterized by HPLC, and were stable in aqueous solution as with **^99m^Tc1,** which contains the bulky PNP3—though they were more vulnerable to transchelation reactions. Actually, the three [^99m^Tc][Tc(N)(PNP)] synthons allow the efficient preparation of the corresponding monocationic compounds under physiological conditions. H_3_NS-cRGDfK was labeled in aqueous solution (for **^99m^Tc1–2,** the amount of ethanol was 6.66%) and under diluted conditions ranging from 3.35 × 10^−5^ to 7.07 × 10^−5^ M. However, differences in the reaction rate and radiolabeling efficiency were recorded. These were due to the different nucleophilicity and sterical hindrance of PNP ligands, which impacted on their reactivity and different geometrical arrangement around the [Tc≡N]^2+^ core, as disclosed by previously reported computational studies [19]. Geometries of the three [Tc(N)Cl_2_(PNP)] species as optimized by density functional calculations provide a clear explanation of the different reactivity of the activated intermediate complexes toward the nucleophilic attack by a BFCA [19].

Thus, the first effect of the use of the less encumbered and water-soluble PNP is an enhanced reactivity of the corresponding framework toward the cysteine peptide, thus permitting the effective labeling at room temperature. Consequently, [^99m^Tc][Tc(N)(PNPOH)]- is the most performing synthon.

The second effect is related to the lipophilicity and stability of the radioconjugates. The replacement of the bulky PNP3 ligand with tinier and/or hydrophilic bisphosphinoamines such as PNP43 and PNP3OH leads to the formation of more hydrophilic compounds. **^99m^Tc3** was found to possess the higher hydrophilic properties of the series produced by the presence of hydroxymethyl substituents on the P atoms of the PNP ligand. It shows the lower Log P and HPLC retention time and the lowest value of plasma protein binding (7.91%), which is the same magnitude order of RDG labeled with [^99m^Tc]Tc-HYNIC or [^99m^Tc]Tc-tricarbonyl approaches [23,40] (≤5%) and [^18^F]F-Galacto-RGD [46].

**^99m^Tc1–3** are stable in solution under conditions compatible with in vivo administration. In addition, it has been proved that **^99m^Tc1–3** challenge stabilities in vitro depend on PNP. The most hydrophilic **^99m^Tc3** compound bearing the *N*,*N*-bis(di-hydroxymethylenphosphinoethyl)methoxyethylamine (PNP3OH) is more prone to cysteine challenge with respect to the higher lipophilic analogues comprising PNP43 or the bulky PNP3, suggesting that the inertness is influenced by the chemical properties of PNP (basicity and steric factor). Nevertheless, in spite the tendency of **^99m^Tc3** to in vitro undergo transchelation reaction, data from in vitro and in vivo metabolism studies show a good inertness and negligible degradation. Indeed, all compounds proved to be suitably stable after incubation for long periods in biological fluids (sera and murine homogenates) and in vivo. These outcomes confirm the ability of the cysteine chelator to bind and stabilize the [^99m^Tc][Tc(N)(PNPn)] moiety, invariably from bisphosphinoamine usage, avoiding transmetallation to serum proteins or amino acids as well as re-oxidation of the metal to Tc(VII), and prove the radiopeptide resistance to proteolytic degradation by endogenous enzymes.

The third effect is connected with the influence of the [^99m^Tc][Tc(N)(PNP)] framework on the targeting properties of the bioactive peptide, which was confirmed in vitro and in vivo. Cellular studies clearly show the capability of **^99m^Tc1–3** to bind both α_v_β_3_ and α_v_β_5_ receptors, with small differences, suggesting that the affinity for the target is in all cases preserved upon conjugation to the differently sterically crowded building block. Indeed, the radioconjugates present a good level of internalized product in α_v_β_3_- and α_v_β_5_-positive cell lines (M21 and MCF7); internalization significantly decreased in receptor-negative M21L cells and was strongly inhibited in blocking studies when the receptor was completely saturated with the unlabeled cRGDfK peptide. The receptor binding and internalization values are comparable with that of **^99m^Tc**-HYNIC-cRGDfK. **^99m^Tc1** possesses the highest uptake of the series but also the higher unspecific interaction, probably due to the lipophilic character and steric hindrance of PNP3; **^99m^Tc2**, which contains the less encumbered PNP43, displays lower uptake and internalization properties, whereas **^99m^Tc3**, owing to its low lipophilic character and shape, shows the higher internalized activity, followed by **^99m^Tc1** and **^99m^Tc2**.

With respect to the pharmacokinetic and pharmacological profile of the conjugates, in general, all studied compounds have a good distribution profile with rapid blood clearance, mainly through the kidneys, with low hepatobiliary excretion. Organs without expression of integrin such as heart, lung, spleen, and stomach showed low uptake and short MRT (<2.1 min). Despite the high hydrophilic character of **^99m^Tc3**, its liver uptake was similar to that observed for **^99m^Tc1** and **^99m^Tc2**, and a slower excretion rate was observed. However, data from the melanoma model clearly showed that **^99m^Tc3** exhibits the highest uptake in α_v_β_3_ integrin-expressing tumors, and consequently the highest target-to-non-target ratio, possessing the best properties with respect to **^99m^Tc1** and **^99m^Tc2**.

## 4. Materials and Methods

**General**. All chemicals and reagents were purchased from Sigma-Aldrich (Milan, Italy). The aqueous solutions were prepared using milli-Q water (18.2 MΩ·cm ionic purity) obtained with a Milli-Q water system by Millipore (Bedford, MA, USA).

The pentapeptide cRGDfK used in inhibition studies was purchased from PolyPeptide Laboratories (Strasbourg, France).

Sep-Pak RP-C18 and OASIS HLB extraction cartridges were purchased from Waters Corporation (Milford, MA, USA).

Bis-diphosphinoamine ligands were synthesized according to the reported procedure [19].

Due to the tendency of the diphosphinoamine ligands to oxidize, all solvents used in the reactions with PNP were previously degassed to remove the dissolved traces of dioxygen.

Technetium-99m as [^99m^Tc]NaTcO_4_ was eluted from a ^99^Mo/^99m^Tc DryTec-generator provided by GE Healthcare (Milan Italy). **^99m^Tc**-HYNIC-cRGDfK was prepared according to the literature [23].

**Analysis:** Electrospray mass spectrometry (ESI-MS) was performed on API-TOF Mariner Spectrometer (Thermo Finnigan, San Jose, CA, USA) operating in positive ion mode.

High-performance liquid chromatography (HPLC) analyses were used to evaluate the radiochemical yield (RCY) and the stability evaluated as radiochemical purity (RCP) of the compounds. HPLC was performed on a Beckman System Gold instrument equipped with a programmable solvent Model 126, a scanning detector Module 166 (λ = 215 µm), and a radioisotope detector Model 3200 Bioscan. HPLC analysis was performed on a Reverse Phase Vydac 218TP C18 precolumn (5 µm, 4,6 × 45 mm) and Vydac 218TP C18 column (5µm, 4.6 × 250 mm). Flow rate of 1 mL/min. The results and the analysis conditions are reported in Table 1.

**Ligand Synthesis**

Synthesis of the H_3_NS-cRGDfK peptide

Peptide was prepared by SPPS starting from Fmoc-L-Asp(Wang-Resin)-OAll. Peptide was assembled using the Fmoc/HBTU chemistry in 0.06 mmolar scale by manual solid-phase. HBTU/HOBt activation employed a three-fold molar excess (0.18 mmol) of Fmoc amino acids in DMF solution for each coupling cycle. Coupling time was 40 min. Fmoc deprotection was performed with 20% piperidine. The C-terminal allyl protecting group was removed under neutral conditions with a catalytic amount of Pd (PPh_3_)_4_ in the presence of PhSiH_3_ in an argon atmosphere. After on-resin cyclization, the ivDde protecting group was removed from the Lys side chain by treatment with a 2% hydrazine hydrate solution in DMF (3 × 15 min).

After deprotection of ivDde group from the lysine residue, Boc-Cys(Trt)-OH (84.14 mg) was condensed to the ε-amino group of residue. The crude peptide was obtained by treatment with TFA/TIS/EDT/H_2_O (94/1/2.5/2.5) solution followed by precipitation with diethylether, obtaining 48.63 mg of product (yield 57.41%).

The product was purified by HPLC using the following chromatographic conditions: Reverse Phase Vydac C18 218TP1022 column (10 µm, 300 Å, 250 × 22 mm); UV, λ = 215 nm. Flow rate = 12 mL/min. Solvents: A) 0.05% TFA in H_2_O; B) 0.05% TFA in 9:1 *v*/*v* CH_3_CN/H_2_O. ^1^gradient: 0–3 min, %B = 15; 3–28 min, %B = 35; 28–29 min, %B = 90; 29–31 min, %B = 90, 31–32 min, %B = 15. Lyophilization of the collected fraction (R_T_ = 13.0 min) yielded the expected product 18.31 mg. Yield = 37.65%. Upon purification, the chemical purity of the peptide as determined by HPLC was 95%.

ESI-MS: calc. for C_30_H_45_N_10_O_8_S M = 706.32, found m/z = 707.3 for [M+H]^+^ (abundance 100%).

**Radiosynthesis**

**Preparation of** **[^99m^Tc(N)(H_2_NS-cRGDfK)(PNP3,43)** **]^+^ (^99m^Tc1 and ^99m^Tc2)**

[^99m^Tc]NaTcO_4_ (1.0 mL, 50.0 MBq–3.0 GBq) was added to a vial containing succinic dihydrazide (SDH; 2.5 mg) and tin chloride (SnCl_2_, 0.1 mg suspended in 0.1 mL of saline). The vial was kept at room temperature for 15 min, giving a mixture of ^99m^Tc-nitrido precursors [^99m^Tc][Tc≡N]^2+^_int_. Then, PNP (PNP3, 0.481 mg in 0.100 mL of ethanol; PNP43, 0.250 mg in 0.100 mL of ethanol), H_3_NS-cRGDfK (0.025 mg dissolved in 0.100 mL of water), and 0.200 mL of phosphate buffer (0.2 M pH 7.4) were added. The reaction mixture was heated at 80 °C for 30 min. The pH of the reaction mixture, measured at the end of the reaction, was 7. The final volume was 1.5 mL. RCYs as determined by HPLC chromatography are summarized in Table 1. HPLC profiles are shown in Figure 2.

**[^99m^Tc(N)(H_2_NS-cRGDfK)(PNP3OH)]^+^ (^99m^Tc3)**

*Two-step preparation*. To ^99m^Tc-nitrido precursors [^99m^Tc][Tc≡N]^2+^_int_ prepared as above, PNP3OH (0.482 mg dissolved in 0.300 mL of phosphate buffer 0.2 M pH 7.4) and H_3_NS-cRGDfK (0.020 mg dissolved in 0.100 mL water) were added, and the reaction mixture was incubated at room temperature for 30 min. The pH of the reaction mixture, measured at the end of the reaction, was 7. The final volume was 1.5 mL. RCY as determined by HPLC chromatography is summarized in Table 1.

*One-step preparation*. To a vial containing SDH (1 mg) and SnCl_2_ (0.100 mg suspended in 0.100 mL of saline) was added Na[^99m^TcO_4_] (0.500 mL; 50.0 MBq–2.2 GBq). The vial was vortexed for 10 s. Rapidly, PNP3OH⋅(0.482 mg in 0.200 mL of PB 0.3 M, pH 7.4) and H_3_NS-cRGDfK (0.025 mg dissolved in 0.100 mL water) were added. The reaction mixture was left standing at RT for 30 min. The pH of the reaction mixture, measured at the end of the reaction, was 7.2, and the volume 1 mL. HPLC profile is shown in Figure 2.

**Radiolabeling efficiency**. The radiolabeling efficiency of the H_3_NS-cRGDfK ligand was determined following for each compound the standardized two-step procedure, reported above, by using the fixed amount of diphosphinoamine ligand 9.96 10^−4^ mmol. The final volume was 1.5 mL. The reaction time was 30 min.

The concentration of the H_3_NS-cRGDfK ligand was progressively decreased in the range 50 µg–7.5 µg. For **^99m^Tc1** and **^99m^Tc2**, radiosyntheses were conducted both at 80 °C and room temperature, while for **^99m^Tc3**, the incubation was accomplished at room temperature. The RCYs were determined by HPLC.

**Dose preparation.** For biological studies, the radiolabeled peptide was purified from excess reagents using the following procedure.

A reversed-phase C18 cartridge (Sep-Pak, Waters) was preconditioned with ethanol (5.0 mL) followed by deionized water (5.0 mL). The selected compound was diluted with 9.0 mL of water, withdrawn from the reaction vial, and loaded on the cartridge. Approximately 95% of the initial activity was retained on the cartridge.

**^99m^Tc1 and ^99m^Tc2.** After washing the cartridge with saline (20.0 mL), ethanol 15% (5.0 mL), and ethanol 20% (5.0 mL), the complex was eluted using an 40/60 mixture of ethanol/saline (1.5 mL).

***^99m^*Tc3.** After washing the cartridge with saline (20.0 mL) and ethanol 15% (5.0 mL), the complex was eluted using an 40/60 mixture of ethanol/saline (1.5 mL).

Ninety percent of the loaded activity was collected.

In sequence, a cation-exchange cartridge (Sep-Pak, Waters) was preconditioned with deionized water (10.0 mL). The radioactive solution collected from the first purification step was diluted with 9.0 mL of water, withdrawn with a syringe (10.0 mL), and loaded on the cartridge. All activity was retained on the cartridge. The cartridge was washed with deionized water (10.0 mL), ethanol 70% (5.0 mL), ethanol 90% (5.0 mL), and absolute ethanol (5.0 mL). The activity was totally eluted using a 40/60 mixture of ethanol/saline (1.0 mL).

After purification, the complex was diluted in PBS to obtain an isotonic solution containing 5% (*v*/*v*) ethanol and used for in vitro and in vivo studies.

**Determination of log P values.** n-Octanol/PBS partition coefficients were determined by vortex mixing (20 min) 3.0 mL of n-octanol, 3.0 mL of PBS and 100 µL of the radiolabeled compound purified as above. After centrifugation (3000 g for 10 min), aliquots (100 µL) of both the organic and the aqueous phases were collected and counted with a γ-counter.

**In vitro studies**

**Cysteine (Cys) and Glutathione (GSH) Challenge.** Challenge experiments were carried out with the purified complex using an excess of Cys or GSH. An aliquot (50 µL) of an aqueous stock solution of cysteine hydrochloride (10 mM or 2 mM) was added to a propylene test tube containing PBS (400 µL, pH 7.4) and the relevant [^99m^Tc(N)(H_2_NS-cRGDfK)(PNP)]^+^ complex (50 µL). The mixture was vortexed and incubated at 37 °C for 24 h. A control reaction containing an equal volume of water, instead of cysteine hydrochloride, was studied in parallel. At 0.5, 1, 3, and 24 h, aliquots of the reaction mixture were withdrawn and analyzed by HPLC chromatography. A similar procedure was applied using GSH (50 µL, 10 mM) as challenger.

**In vitro metabolism**. The in vitro resistances to the biodegradation process of [^99m^Tc(N)(H_2_NS-cRGDfK)(PNP)]^+^ were evaluated by monitoring the variation in RCP of the complexes over time after incubation in different milieu. In a propylene test tube, 50 µL of purified compound was added to 450 µL of: PBS (pH = 7.4); human or rat serum; rat kidney or liver homogenates. The resulting mixture was incubated at 37 °C for 4 h.

For incubation with kidney and liver homogenates, the organs freshly excised from rats were rapidly rinsed, diluted to 30/*p*/*v* with HEPES buffer (20 mM; pH 7.2), and homogenized in an Ultra-Turrax T25 homogenator for 1 min at 4 °C. The radiopeptides were incubated with fresh homogenates at 37 °C.

At 0.25, 0.5, 1, 2, and 4 h, 50 µL of each solution were withdrawn, diluted with of phosphate buffer (950 µL 0.02 M, pH 7.4), and treated with OASIS HLB extraction cartridge in accordance with the suppliers’ instructions. The activity was eluted with a mixture of ethanol/saline 60% (1 mL). Eighty percent of the initial activity was collected in the elution fraction. Degradation of the complexes was assessed by HPLC. The experiments were performed in duplicate.

**Protein Binding.** The affinity of the ^99m^Tc(N) complexes toward the serum proteins was evaluated by chromatographic methods.

Upon incubation, 25 µL of each sample was withdrawn and loaded on a pre-spun (735× *g* for 2 min) Sephadex G-50 mini-column. The column was centrifuged at 735 × *g* for 1 min. The collected eluate and the column were counted in a NaI-scintillation counter. The protein-bound complex was calculated as the percentage of the total activity.

**Cell Studies**

Human melanoma M21 (α_v_β_3_ positive) and the integrin-negative counterpart M21-L cells were kindly provided by Prof. Decristoforo (Innsbruck Medical University, Austria). Human breast adenocarcinoma MCF7 cells (α_v_β_5_ positive) were purchased from the American Type Culture Collection (ATCC, Manassas, VA, USA).

Cells were cultured in in RPMI 1640 (EuroClone, Milan, Italy) supplemented with 10% (*v*/*v*) heat-inactivated fetal bovine serum (Gibco BRL, Paisley, UK), 2 mM l-glutamine (Lonza, Verviers, Belgium), 10 mM HEPES (Lonza), 100 U/mL penicillin/streptomycin (Lonza). Cell lines were maintained at 37 °C in a humidified atmosphere containing 5% CO_2_.

Qualitative and quantitative expression of α_v_β_3_ and α_v_β_5_ integrins on cancer cell lines was investigated by flow cytometry [11].

Before the cell experiments, the stability of **^99m^Tc1–3** was evaluated by incubating the purified complexes in RPMI1640 medium at 37 °C for 2h. Thus, to a glass test tube containing the cell culture media (990 µL), the radioconjugate (10 µCi in 10 µL of PBS) was added. The mixture was vortexed and incubated. At 0.5, 1, and 2 h, 100 µL of the reaction mixture was withdrawn and analyzed by HPLC. RCPs were found >90%.

Experiments were conducted in suspension at 37 °C. The working conditions were set up to avoid cell damage and/or death. With the use of these information, the cells were pre-incubated in sterile glass tubes at 37 °C for 10 min, in the presence or not of cRGDfK pentapeptide (50 µL; 1.65 mM).

Then, 1 mL of the cell suspension (1 × 10^6^ cells/mL) was incubated with intermittent agitation with 10 µL (10 µCi) of **^99m^Tc1–3**. At 90 min, the cells were moved in Eppendorf microcentrifuge tubes (1.5 mL) and the incubation was interrupted by centrifugation (3000 rpm for 5 min at 10 °C). The supernatant (S1) was separated from the pellet. The cell pellet was treated 10 min at room temperature with saline acetate buffer (CH_3_COOH 0.2M/NaCl 0.5 M) to remove the membrane-bound compound. After centrifugation (3000 rpm for 10 min at 10 °C), the supernatant (S2) was separated from the pellet (P1), and each fraction was collected, placed in a separated counting tube, and the activity was measured using a NaI counter (Cobra II, Packard) to estimate both the cell uptake and the internalized fraction.

The uptake of complexes was expressed as percentage cell uptake of the total activity on 10^6^ cells (% cell uptake/10^6^ cells).

All assessments were conducted in triplicate for three experiments.

To evaluate whether **^99m^Tc1–3** are stable upon cell exposure, 100 µL of S1 was analyzed by HPLC. All eluted radioactivity was found to overlap the native [^99m^Tc(N)(PNP)]-tagged peptide.

**Animal Studies**

*Pharmacokinetics studies in healthy rats and in vivo metabolism*

Female Sprague Dawley rats weighing 180–200 g were anesthetized with an intraperitoneal injection of a mixture of Zoletil (40 mg/kg) and xylazine (2 mg/kg). A jugular vein was surgically exposed, and the solution containing the radiolabeled peptide was injected (15 µCi/100 µL). The rats (n = 3) were scarified by cervical dislocation at different times after injection. The blood was withdrawn from the heart with a syringe immediately after death and quantified. Organs were excised, rinsed in saline, weighed, and the activity was counted in a NaI counter. The results were expressed as the percentage of injected activity per gram of tissue (%IA/g) to be able to compare our results with those reported in the literature.

The time–activity curves of the main organs were obtained using the biodistribution data (%IA/organ) as input for the CoKiMo spreadsheet software [35]. Then, the organ mean residence times (MRT) were computed by integration of the organ activity curves, extrapolated until 5 half-lives of Tc-99m (30 h) and corrected for its physical decay, to evaluate the pharmacokinetic profile of each radiolabeled peptide.

*Metabolites in the urine*. At 2 and 4 h p.i., urine was collected directly from the rat bladder and analyzed by HPLC using method 2 described in Table 1.

*Biodistribution in tumor-bearing mice*

Human melanoma M21(α_v_β_3_-positive) were injected subcutaneously (s.c. 1 × 10^6^ cells) in the right flank of six week-old male NSG mice (Charles River Laboratories). As a negative control, M21L (α_v_β_3_-negative) were inoculated s.c. (1 × 10^6^ cells) in the left flank of the same mice. Fifteen days after the inoculation, when the tumors are palpable (5–3 mm), the mice were injected in the tail vein with the selected radiolabeled peptide (20 µCi/100 µL of PBS). A group of six animals was sacrificed at 120 min p.i. by CO_2_ inhalation. A sample of blood was collected and tumors and normal tissues of interest were dissected, rinsed to remove the excess of blood, weighted, and their radioactivity measured using a NaI counter. Results are expressed as the percentage of injected activity per gram (% IA/g) and tumor-to-organ ratios were calculated.

## 5. Conclusions

In this study, we assessed and compared, for the first time, the effects of chemical-physical properties of three different [^99m^Tc][Tc(N)(PNP)] synthons on the synthesis and biological fate of a small [^99m^Tc][Tc(N)(PNP)]-labeled peptide to identify the best performing synthon useful in preparation of target specific compounds.

Variation in the nature of the PNP has a profound impact on the overall chemical and physical properties of the bio-conjugated complex. All compounds are equally synthetically accessible and easy to purify and administer. Notably, the use of PNP3OH allows the labeling of the peptide at room temperature without significantly reducing the labeling efficiency or stability of the construct.

The main influences of the synthon on the targeting peptide were observed in the in vitro cell binding and in vivo. In healthy and xenograft animal models, different pharmacokinetics and tumor accumulation were observed as a function of the lipophilicity and sterical hindrance of the synthon: **^99m^Tc3** and **^99m^Tc1** shared almost the same high tumor uptake and similar distribution properties, meanwhile a reduced performance of **^99m^Tc2** was evident.

By considering the overall data, the *ws*[^99m^Tc][Tc(N)(PNP3OH)]–, and [^99m^Tc][Tc(N)(PNP3)]– are better performing synthons in terms of efficiency and biological profile than the [^99m^Tc][Tc(N)(PNP43)]– building block, and consequently more suitable for further radiopharmaceutical applications.

Furthermore, the good labeling properties of *ws*[^99m^Tc][Tc(N)(PNP3OH)]– framework can be exploited to extend this technology to the labeling of temperature-sensitive biomolecules suitable for SPECT imaging.

## Figures and Tables

**Table 1:** RCY of ^99m^Tc1–3, along with their chromatographic properties.

Complex			HPLC Rt min	a/b	%RCY	% Protein Binding *	Log P
H_3_NS-cRGDfK			13.00 ^1^				
[Tc(N)(H_2_NS-cRGDfK)(PNP3)]^+^	**^99m^Tc1**	a	14.09 ^1^; 11.39 ^2^	61.76/31.98	93.74 ± 0.05	14.95 ± 1.95	−1.72 ^1^
		b	15.79 ^1^; 13.23 ^2^				
[Tc(N)(H_2_NS-cRGDfK)(PNP43)]^+^	**^99m^Tc2**	a	12.78 ^2^; 26.04 ^3^	60.64/27.80	89.04 ± 0.09	13.63 ± 2.95	−1.95
		b	15.45 ^2^; 29.08 ^3^				
[Tc(N)(H_2_NS-cRGDfK)(PNP3OH)]^+^	**^99m^Tc3**	a	8.21 ^2^; 22.75 ^3^	67.56/23.33	90.89 ± 0.11	7.91 ± 2.01	−2.39
		b	10.12 ^2^; 24.86 ^3^				
[Tc-HYNIC-cRGDfK]			9.02 ^2^			2.3 ± 0.11	−3.52

* % protein binding measured at 1h after incubation. HPLC analysis was performed on a Reverse Phase Vydac 218TP C18 precolumn (5 µm, 4.6 × 45 mm) and Vydac 218TP C18 column (5 µm, 4.6 × 250 mm). Flow rate of 1 mL/min. UV λ = 216. Solvents: A H_2_O TFA 0.05%; B CH_3_CN TFA 0.05%. ^1^ gradient: 0–1 min, %B = 25; 1–17 min, %B = 32; 18–23 min, %B = 32; 23–24 min, %B = 90; 24–28 min, %B = 90; 28–29 min, %B = 25; 29–30 min, %B = 25. ^2^ gradient: 0 min, %B = 15; 1–11 min, %B = 22; 11–21 min, %B = 22; 21–22 min, %B = 90; 22–27 min, %B = 90; 27–28 min, %B = 15; 28–30min, %B = 15; ^3^ gradient: 0 min, %B = 5; 3–28 min, %B = 25; 28–29 min, %B = 80; 29–32 min, %B = 80; 32–34 min, %B = 5; 34–35 min, %B = 5.

**Table 2:** Variation in integrins expression on the tumor cell lines used in our experiments.

Cell Line (Tumor Type)	ΔMFI α_v_β_3_	α_v_β_3_	ΔMFI α_v_β_5_	α_v_β_5_
M21 (Human melanoma)	25	+++	1	----
MCF7 (Human breast cancer)	4	----	67	++++
M21-L (Human melanoma)	1	----	0	----
Range for MFI values of α_v_β_3_	Range for MFI values of α_v_β_5_			
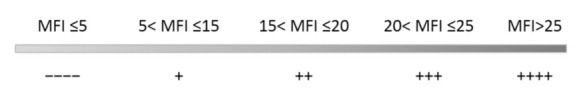	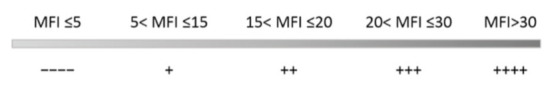			

ΔMFI is calculated by subtracting the MFI value of the autofluorescence sample (cells alone) from the MFI value of the sample test (cells incubated with anti-α_v_β_3_ or α_v_β_5_ antibodies): ΔMFI = MFI(test) − MFI(autofluorescence). Ranges for MFI values are arbitrarily selected to classify cell lines according to their integrin expression.

**Table 3:** Biodistribution studies of the [^99m^Tc(N)(H_2_NS-cRGDfK)(PNPn)]^+^ complexes in healthy female Sprague Dawley rats (*n* = 4).

	^99m^Tc1				^99m^Tc2				^99m^Tc3			
Organs	30 min	60 min	120 min	240 min	30 min	60 min	120 min	240 min	30 min	60 min	120 min	240 min
Blood	0.81 ± 0.05	0.30 ± 0.04	0.09 ± 0.01	0.05 ± 0.01	0.65 ± 0.04	0.35 ± 0.07	0.19 ± 0.04	0.11 ± 0.02	0.46 ± 0.07	0.25 ± 0.03	0.16 ± 0.01	0.09 ± 0.01
Heart	0.26 ± 0.05	0.15 ± 0.01	0.10 ± 0.02	0.08 ± 0.01	0.30 ± 0.01	0.20 ± 0.04	0.15 ± 0.06	0.12 ± 0.01	0.25 ± 0.06	0.15 ± 0.00	0.13 ± 0.02	0.12 ± 0.03
Lungs	0.64 ± 0.09	0.43 ± 0.01	0.25 ± 0.03	0.16 ± 0.04	0.82 ± 0.09	0.56 ± 0.10	0.42 ± 0.02	0.34 ± 0.04	0.62 ± 0.08	0.49 ± 0.01	0.37 ± 0.06	0.32 ± 0.02
Liver	0.96 ± 0.10	0.75 ± 0.02	0.68 ± 0.06	0.66 ± 0.07	0.90 ± 0.13	0.73 ± 0.01	0.57 ± 0.03	0.52 ± 0.07	0.90 ± 0.09	0.80 ± 0.06	0.75 ± 0.09	0.67 ± 0.04
Spleen	0.65 ± 0.09	0.51 ± 0.04	0.47 ± 0.04	0.45 ± 0.00	0.47 ± 0.02	0.40 ± 0.05	0.38 ± 0.04	0.34 ± 0.07	0.43 ± 0.05	0.39 ± 0.04	0.36 ± 0.02	0.34 ± 0.04
Kidneys	2.71 ± 0.18	1.57 ± 0.13	0.92 ± 0.20	0.88 ± 0.09	6.77 ± 0.86	6.20 ± 0.68	5.51 ± 0.42	4.27 ± 0.31	5.22 ± 0.44	4.83 ± 0.35	4.44 ± 0.32	4.22 ± 0.45
Stomach	0.53 ± 0.07	0.41 ± 0.02	0.34 ± 0.04	0.29 ± 0.02	0.82 ± 0.07	0.64 ± 0.08	0.51 ± 0.08	0.41 ± 0.09	0.72 ± 0.04	0.64 ± 0.08	0.57 ± 0.03	0.46 ± 0.04
Intestine	0.65 ± 0.16	0.60 ± 0.12	0.40 ± 0.07	0.38 ± 0.01	1.19 ± 0.14	1.69 ± 0.17	1.94 ± 0.25	1.90 ± 0.19	1.29 ± 0.13	1.95 ± 0.17	3.76 ± 0.27	4.17 ± 0.59
Muscle	0.21 ± 0.09	0.13 ± 0.06	0.10 ± 0.06	0.06 ± 0.01	0.22 ± 0.03	0.14 ± 0.03	0.12 ± 0.01	0.09 ± 0.00	0.17 ± 0.01	0.13 ± 0.01	0.12 ± 0.00	0.08 ± 0.00
Urine	27.2 ± 11.7	33.1 ± 5.6	54.3 ± 1.9	55.9 ± 7.1	17.7 ± 3.6	28.5 ± 4.6	41.5 ± 4.5	43.5 ± 3.4	27.8 ± 5.6	31.2 ± 3.0	44.2 ± 10.9	30.2 ± 9.45

Data are expressed as %IA/g (*n* = 4). Activity for stomach and urine are expressed as %IA.

**Table 4:** Mean resident time (MRT) of **^99m^Tc1–3** complexes in the main organs.

Organ	MRT (min)		
	^99m^Tc1	^99m^Tc2	^99m^Tc3
Blood	14.7	16.9	14.5
Heart	0.3	0.4	0.4
Lung	0.9	1.6	1.6
Liver	30.5	25.9	26.7
Kidney	9.2	30.7	36.7
Spleen	1.2	0.8	0.8
Stomach	1.5	2.1	2.1

**Table 5:** Biodistribution studies at 120 min p.i. of **^99m^Tc1**, **^99m^Tc2**, **^99m^Tc3** in tumor-bearing mice (n = 5).

Organs	^99m^Tc1	^99m^Tc2	^99m^Tc3	^99m^Tc_HYNIC [40]	
				1 h	4 h
Blood	0.07 ± 0.02	0.12 ± 0.03	0.05 ± 0.01	0.98 ± 0.04	0.45 ± 0.02
Heart	0.47 ± 0.03	0.48 ± 0.07	0.44 ± 0.09	1.01 ± 0.05	0.64 ± 0.09
Lungs	1.25 ± 0.23	1.04 ± 0.08	1.32 ± 0.20		
Liver	3.59 ± 0.31	2.43 ± 0.23	3.22 ± 0.24	2.63 ± 0.14	1.71 ± 0.28
Spleen	1.61 ± 0.01	1.26 ± 0.04	1.43 ± 0.19		
Kidneys	4.10 ± 0.37	3.38 ± 0.36	3.34 ± 0.18	3.68 ± 0.13	2.81 ± 0.37
Stomach *	1.22 ± 0.15	1.02 ± 0.16	1.05 ± 0.14	2.50 ± 0.75	1.28 ± 0.22
Intestine	2.30 ± 0.10	2.45 ± 0.29	3.09 ± 0.62	2.04 ± 0.18	1.29 ± 0.25
Muscle	0.37 ± 0.04	0.31 ± 0.05	0.23 ± 0.03	0.76 ± 0.69	0.34 ± 0.05
M21	3.17 ± 0.60	1.08 ± 0.24	3.92 ± 0.69	2.73 ± 0.26	2.06 ± 0.37
M21L	1.73 ± 0.19	0.42 ± 0.05	0.70 ± 0.16	0.85 ± 0.20	
M21/M21L	1.83	2.57	5.60	3.21	
M21/blood	29.96	11.26	73.42	2.79	4.58
M21/muscle	9.15	3.54	16.72	3.59	6.05
M21/liver	0.88	0.44	1.11	1.03	1.20
M21/lung	2.53	1.04	2.98		
M21/kidneys	0.77	0.32	1.23	0.74	0.73

Data are expressed as %IA/g. * Data are expressed as %IA.

## Data Availability

Not applicable.

## Supplementary Materials

The following supporting information can be downloaded at: https://www.mdpi.com/article/10.3390/molecules27082548/s1, Figure S1. HPLC profile and ESI-MS spectrum of the pure H_3_NS-cRGDfK

## Sample Availability

Samples of the Bis-phosphinoamine (PNP) compounds are available from the authors.
