# Supplementary material for: Impact of Different [Tc(N)PNP]-Scaffolds on the Biological Properties of the Small cRGDfK Peptide: Synthesis, In Vitro and In Vivo Evaluations
Source: Molecules. 2022 Apr 14;27(8):2548. doi: 10.3390/molecules27082548 (PMC9029856; doi:10.3390/molecules27082548)
Supplement: Supplementary file 1 [file molecules-27-02548-s001.zip › molecules-1667908-supplementary.pdf]

Article

Impact of Different [Tc(N)PNP]−scaffolds on the Biological Properties of the Small RGDfK Peptide: Synthesis, in Vitro and in Vivo Evaluations.

Supplementary

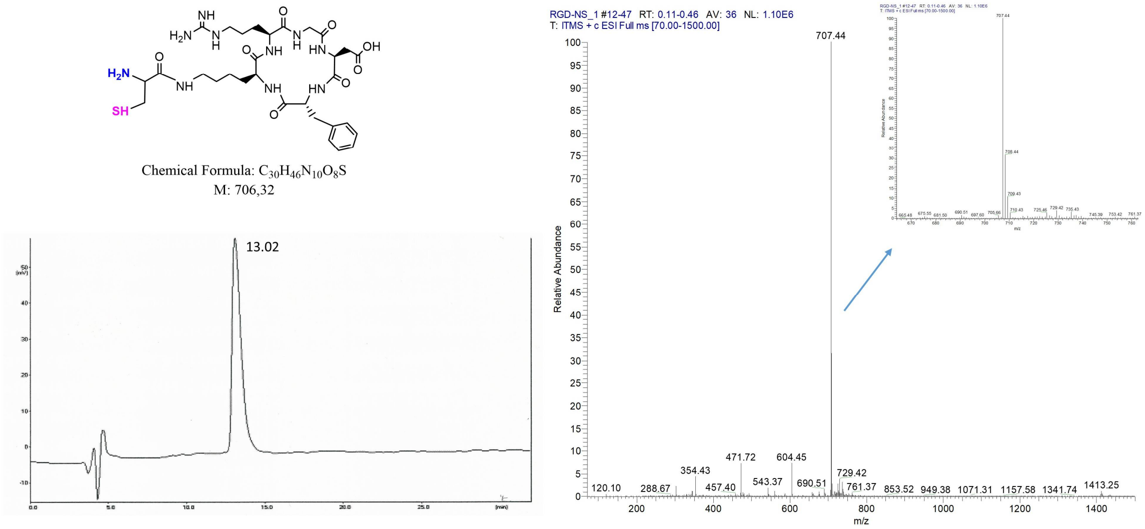

Figure S1. HPLC profile and ESI-MS spectrum of the pure H_3NS-RGD
